# Identification of Key Genes and Pathways Associated With Gender Differences in Pulmonary Arterial Hypertension Based on Bioinformatic Approaches

**DOI:** 10.1155/bmri/9938078

**Published:** 2026-03-14

**Authors:** Mohammad Panahi, Reyhaneh Kalhor, Mohammad-Hossein Mokhtarian, Fatemeh Javaheri Tehrani, Zinat Shams, Hourieh Kalhor

**Affiliations:** ^1^ Centre for Pulmonary Hypertension, Thoraxklinik Heidelberg GmbH at Heidelberg University Hospital, Heidelberg, Germany; ^2^ Laboratory for Molecular Genetic Diagnostics, Institute of Human Genetics, University of Heidelberg, Heidelberg, Germany, uni-heidelberg.de; ^3^ Cellular and Molecular Research Center, Qom University of Medical Sciences, Qom, Iran, muq.ac.ir; ^4^ Department of Clinical Sciences, Faculty of Veterinary Medicine, Garmsar Branch, Islamic Azad University, Garmsar, Iran, azad.ac.ir; ^5^ Department of Biological Science, Kharazmi University, Tehran, Iran, khu.ac.ir

**Keywords:** differentially expressed genes, Gene Ontology, hub gene, protein–protein interaction, pulmonary arterial hypertension

## Abstract

Pulmonary arterial hypertension (PAH) is a complex disease with multiple contributing factors. Epidemiological data showed that women are more susceptible to PAH, but they tend to have better right ventricular (RV) function and prognosis compared to men. The mechanisms behind these gender differences are not well understood. Using a variety of bioinformatic techniques, this study was aimed at investigating the major genes and putative pathways driving PAH in females. Consequently, microarray datasets GSE38267 and GSE131793 were obtained from the Gene Expression Omnibus (GEO) database. Differentially expressed genes (DEGs) in patients with PAH and healthy men and females were found via the use of volcano plots and Venn diagrams. Female‐specific DEGs were selected for Gene Ontology (GO) and Kyoto Encyclopedia of Genes and Genomes (KEGG) pathway enrichment analyses, with results visualized via R and Cytoscape software. The protein–protein interactions (PPIs) of female‐specific DEGs were analyzed using the NetworkAnalyst online tool. Among identified DEGs, 98 DEGs were specific to males, 145 were shared among sexes, and 741 were unique to females. To focus on DEGs that are specific to female, male and shared DEGs were excluded. GO enrichment analysis revealed that these DEGs in females were mostly involved in cell–matrix adhesion, mucosal innate immune response, and hydrogen peroxide catabolism. Significant KEGG pathways included fluid shear stress, atherosclerosis, arrhythmogenic RV cardiomyopathy, and ECM–receptor interaction. Bioinformatic analysis of microarray datasets led to the identification of 10 female‐specific hub genes, *SLC4A1*, *THBS1*, *ITGB3*, *IL7R*, *CCR7*, *SNCA*, *CTNNB1*, *SELP*, *GZMK*, and *ITGA2B*, from DEGs between control and PAH samples in females. These hub genes that are specific to females have the potential to be significant in the pathogenesis of PAH in females. These hub genes may be promising candidates for improving our knowledge of sex‐related processes in PAH; nevertheless, experimental confirmation is necessary before considering them as biomarkers or therapeutic targets.

## 1. Background

Pulmonary hypertension (PH) refers to a constellation of health conditions that display a gradual increase in pulmonary vascular resistance and pulmonary arterial pressure, which eventually results in the development of right heart failure and mortality [1]. Pulmonary arterial hypertension (PAH) results from substantial irregularities in the precapillary pulmonary vascular system. This illness is characterized by a chronic and progressive panvasculopathy that primarily targets the pulmonary vasculature, resulting in an elevated pulmonary vascular load [2]. PAH, as redefined by the 6th World Symposium on PH, is classified as Group 1 PH distinguished by mean pulmonary artery pressure (mPAP) > 20 mm Hg, pulmonary artery wedge pressure (PAWP) ≤ 15 mm Hg, and PVR ≥ 3 WU during a right heart catheterization (RHC) at a state of resting [2].

It has long been established that women have a significantly higher incidence of PAH compared to men [3]. Sex contributes as an immutable risk factor for several diseases [4, 5]; nonetheless, its consequences create challenges in some instances, such as PH. Sex disparities in PH were shown not only in terms of its prevalence but also in terms of its severity, responsiveness to treatment, and rate of survival [6]. Women are at a greater risk of developing PAH than men. However, women typically have a more favorable prognosis once the disease has developed, which can be partially attributed to their superior right ventricular adaptations [7]. Furthermore, the penetrance of PAH disease is determined by sex, with a prevalence of 42% in females compared to 14% in male patients with heritable pulmonary arterial hypertension (HPAH) [8]. Significantly, male patients diagnosed with PAH experience a more pronounced burden, as seen by a roughly 10% decrease in the 5‐year survival rate (53%) in comparison to females (62.9%) [9]. Despite the existence of numerous theories, a comprehensive comprehension of the molecular and cellular mechanisms that underlie the observed sex‐related differences in PAHs remains to be achieved. These frequently involve hormonal modifications, although metabolism, heredity, and/or the immune system may also be involved [4, 10, 11]. Consequently, it is essential to investigate the cellular and molecular mechanisms behind the disparities in PAH across genders. This will enhance our comprehension of these intricate pathways, perhaps aiding in the identification of methods to halt or mitigate PAH progression.

Currently, bioinformatic techniques are often used to evaluate gene sequencing data of different diseases to identify differentially expressed genes (DEGs), along with performing various analyses. To facilitate the repositioning of complex disease mechanisms that are already well‐established, a multitude of databases and advanced online resources were created [12]. There was a growing body of research utilizing microarray technology to study DEGs and their associated molecular functions (MFs), biological processes (BPs), cellular components (CCs), and regulatory pathways in target disease states [13]. Microarray technology is used to identify genome‐wide variations in gene expression between healthy and patient samples. This technique has been extensively utilized in studying the pathobiology of PAH [10, 14].

Two Gene Expression Omnibus (GEO) microarray datasets were assembled for the subsequent studies. DEGs were detected and compared between healthy controls and PAH patients in both males and females. After identifying female‐specific DEGs using gene set enrichment analysis (GSEA), protein–protein interaction (PPI) networks, Gene Ontology (GO) annotation, and Kyoto Encyclopedia of Genes and Genomes (KEGG) pathway enrichment, potential female‐specific hub genes and key signaling pathways associated with PAH in women were evaluated.

## 2. Materials and Methods

### 2.1. Data Collecting and Processing

This work used two microarray datasets (GSE38267 [11] and GSE131793 [10]) that were sourced from the GEO database [12]. These databases were chosen by looking for PAH, microarray, expression profiling by array, and blood samples. With 64 samples, the GSE38267 dataset was based on the GPL13607 platform and included both male (*n* = 25) and female (*n* = 39) patients with PAH and matched healthy controls. In this research, we compared male and female PAH patients to matching healthy controls. On the other hand, the GSE131793 dataset, which was constructed using the GPL6244 platform, consists of 20 samples, all of which are female PAH patients and controls. Since all datasets were completely deidentified and made accessible to the public, our analyses did not need further ethical clearance or informed permission.

The quantile approach from the limma (linear models for microarray data) package [15] in the R software (Version 4.1.1) environment was used to standardize gene expression data.

Each dataset′s raw expression data was separately standardized using the limma package′s quantile normalization approach to minimize any potential batch effects. Since GSE38267 and GSE131793 were produced on distinct microarray platforms, it was not possible or appropriate to perform direct cross‐dataset batch‐effect correction. Instead, we focused on DEGs consistently detected across both female datasets, thereby reducing dataset‐specific bias without introducing cross‐batch artifacts.

### 2.2. Identification of the DEGs

Different packages were used to identify DEGs from the chosen datasets: limma (Version 3.48.3), data table (Version 1.14.2), plyr (Version 1.8.6), BioGenerics (Version 0.40.0), BioBase (Version 2.54), ggplot (Version 3.3.5) in R software (Version 1.1.4), and the Venn diagram tool (https://bioinformatics.psb.ugent.be/webtools/Venn). DEGs were defined for the GSE38267 dataset as genes showing upregulation with a log fold change (logFC) > 1 and downregulation with a logFC ≤ −1, along with an adjusted *p* value < 0.05. Similar upregulation with a logFC > 0.5 and downregulation with a logFC ≤ −0.5 was seen for the GSE131793 dataset, with a significance criterion of an adjusted *p* value < 0.05 being maintained. To account for multiple hypothesis testing, the Benjamini–Hochberg procedure was applied to control the false discovery rate (FDR). Genes with FDR‐adjusted *p* values (*q* < 0.05) were considered statistically significant. This criterion was consistently used for both DEG identification and functional enrichment studies (GO and KEGG). We used different logFC criteria for the two datasets since they had different sample sizes and statistical powers. The GSE38267 dataset (*n* = 64, including both sexes) provided higher statistical robustness, allowing us to use a stringent cutoff (|log2FC| ≥ 1.0) to minimize false positives. In contrast, the GSE131793 dataset (*n* = 20, all females) had fewer samples, and applying the same strict threshold could have led to false negatives and loss of biologically meaningful genes. Therefore, we applied a more relaxed threshold (|log2FC| ≥ 0.5) for GSE131793 to balance sensitivity and specificity. This approach was consistent with integrative bioinformatic practices where dataset characteristics necessitate adaptive thresholds.

### 2.3. Distinct Gene Expression in Females

We used limma with quantile normalization and empirical Bayes moderation to conduct differential expression analysis within each dataset independently (female PAH vs. female controls; male PAH vs. male controls) in order to identify “female‐specific” DEGs (see Section 2.2). DEGs were required to pass FDR control (Benjamini–Hochberg) at *q* < 0.05 and meet dataset‐specific effect‐size thresholds (|log2FC| ≥ 1.0 for GSE38267; |log2FC| ≥ 0.5 for GSE131793; see Section 2.2). A gene was considered female‐specific if it was significantly differentially expressed in females (PAH vs. control) with a consistent direction of effect in both female datasets (GSE38267 female subset and GSE131793) and was not significantly different in males (PAH vs. control) in GSE38267. Genes shared between sexes were excluded. This filtering was operationalized using Venn diagrams across the four sets (female DEGs in each dataset; male DEGs in GSE38267) to ensure that only distinctly female‐associated DEGs were retained for downstream GO/KEGG, PPI, and hub gene analyses.

### 2.4. Pathway Enrichment Analysis of DEGs

Using the enrichR package [16] in R software (Version 4.1.1) and SRplot (a free online platform) [17], the biological activities of DEGs in females were assessed by KEGG pathway analysis and GO annotation. Three functional classes were established for these genes: BPs, MFs, and CCs. Next, we displayed dot plots for GO enrichment analysis and cnetplot for KEGG pathway enrichment analysis. Enrichment *p* values were adjusted for multiple testing using the Benjamini–Hochberg FDR, and terms with adjusted *p* < 0.05 were considered significant.

### 2.5. Hub Genes and Networks of PPIs

DEG complex protein interactions for 58 DEGs in females were studied using PPI network analysis. We examined predicted and known PPIs using the Search Tool for Retrieval of Interacting Genes (STRING) database [18] (https://cn.string-db.org/), which is a database for searching and predicting PPIs. The obtained network was then graphically represented and examined using the Cytoscape (Version 3.7.2) software [19] and the CytoHubba plug‐in [20]. In the plug‐in CytoHubba tool, five methods were used to identify hub genes within the PPI. The approaches included maximum clique centrality (MCC), edge percolated component (EPC), node connectivity degree (degree), maximal neighborhood component (MNC), and node connectivity closeness (closeness). Hub genes were identified by choosing those that were among the Top 10 across all five methods.

## 3. Results

### 3.1. Identification of DEGs in PAH

To identify DEGs that are specifically in females and whose expression remains unchanged in males, both female and male groups from the GSE38267 and GSE131793 datasets were selected; more details are tabulated in Table 1. DEGs were studied using the “limma” package. There were 961 DEGs in GSE38267, including 243 DEGs in PAH males and 718 in PAH females, compared to healthy controls. All of these DEGs passed the FDR threshold (*q* < 0.05), ensuring that the reported results are statistically robust.

**Table 1:** Overview of the analyzed datasets (GSE38267: 12 PAHs and nine controls; GSE131793: 17 PAHs and nine controls). All DEGs were identified using the limma package with a significance threshold of FDR < 0.05.

Dataset	GPL	Platform topic	Country	Submission data	Sample		
					Total	Control	Patient
GSE38267	GPL13607	Agilent‐028004 SurePrint G3 Human GE 8x60K Microarray (Feature Numberversion)	France	2012	41	28	13
GSE131793	GPL6244	[HuGene‐1_0‐st] Affymetrix Human Gene 1.0 ST Array [transcript (gene) version]	United States	2019	20	10	10

In the GSE131793 dataset, 170 DEGs were identified; all samples in this dataset were for females (Figure 1a). All of these DEGs also satisfied the FDR threshold (*q* < 0.05).

Figure 1Volcano plots and Venn diagrams of DEGs for the GSE38267 and GSE131793 datasets. (a) Volcano plots of GSE38267 and GSE131793 datasets. Each dot represents a gene, with the *x*‐axis showing log2 fold change (FC) and the *y*‐axis showing −log10 adjusted *p* value. Genes toward plot edges were highly upregulated (right) or downregulated (left), while those on the plot had lower *p* values, indicating significance. Color and shape may differentiate between the significant genes. (b) Venn diagram showing differentially expressed genes (DEGs). There were 243 and 717 DEGs obtained from GSE38267 for males and females, respectively. Besides, there were 170 DEGs obtained from the GSE131793.

**Figure:**
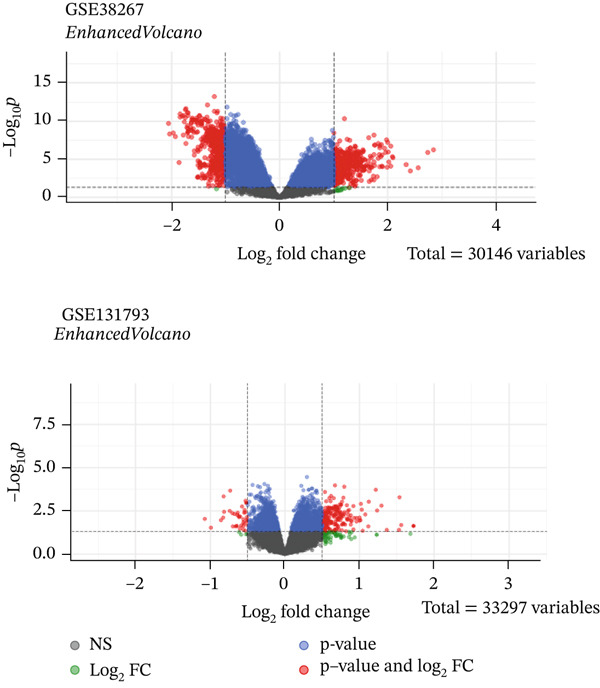
(a)

**Figure:**
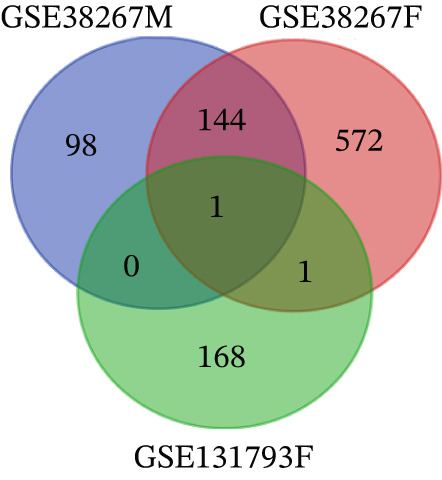
(b)

A Venn diagram was prepared to identify final DEGs in both datasets for males and females separately (Figure 1b). To identify female‐specific DEGs, we excluded the male‐specific (243) and shared DEGs (144) between males and females from our study. Among DEGs in females, based on logFC and *p* values, 34 downregulated and 24 upregulated genes were selected in females. The use of the female‐specific filter, which was significant in both female datasets with concordant direction and nonsignificant in males, resulted in 58 final female‐specific DEGs that were then utilized for enrichment and network analysis.

### 3.2. Functional Enrichment Analysis of DEGs in Females

To evaluate PAH‐related functional annotations and pathway enrichments in females, we selected 58 final DEGs for GO and KEGG analyses. Detailed findings are presented in Tables 2 and 3.

**Table 2:** The Top 10 results for biological processes (BPs), cellular components (CCs), and molecular functions (MFs) from the GO functional annotation analysis of differentially expressed genes (DEGs) in females. The analysis was performed on 58 female‐specific DEGs derived from 26 total samples (17 PAH females and nine female controls) using the limma package with an adjusted *p* value (FDR) < 0.05 as the significance threshold.

ID	Description	Count	Gene ID	*p* adjust	*p* value
GO‐BPs					
GO:0007160	Cell–matrix adhesion	6	*CTNNB1*/*ITGB3*/*ITGA2B*/*THBS1*/TRPM7/*CCR7*	0.017204476	1.47402e − 05
GO:0002227	Innate immune response in the mucosa	3	DEFA1B/LTF/RNASE2	0.017204476	2.96407e − 05
GO:0042744	Hydrogen peroxide catabolic process	3	HP/*SNCA*/PRDX4	0.017204476	5.59955e − 05
GO:0030099	Myeloid cell differentiation	7	*CTNNB1*/ALAS2/*SLC4A1*/*ITGA2B*/LTF/*THBS1*/*CCR7*	0.017204476	5.70985e − 05
GO:0006979	Response to oxidative stress	7	*CTNNB1*/HP/*SNCA*/*CCR7*/PRDX4/NOS3/IL18RAP	0.017204476	8.36185e − 05
GO:0002385	Mucosal immune response	3	DEFA1B/LTF/RNASE2	0.017204476	9.43117e − 05
GO:0032496	Response to lipopolysaccharide	6	DEFA1B/LTF/*SNCA*/*SELP*/*CCR7*/NOS3	0.017204476	0.000117846
GO:0002251	Organ or tissue‐specific immune response	3	DEFA1B/LTF/RNASE2	0.017204476	0.000118595
GO:0051354	Negative regulation of oxidoreductase activity	3	HP/*SLC4A1*/*SNCA*	0.017204476	0.000118595
GO:0043312	Neutrophil degranulation	7	DEFA1B/HP/LTF/RNASE2/PPIA/GAA/PRDX4	0.017204476	0.000122555

GO‐CCs					
GO:0031092	Platelet alpha granule membrane	4	*ITGB3*/*ITGA2B*/*SNCA*/*SELP*	1.03152e − 05	7.42101e − 08
GO:0031091	Platelet alpha granule	5	*ITGB3*/*ITGA2B*/*SNCA*/*SELP*/*THBS1*	0.000198267	2.85277e − 06
GO:0009897	External side of the plasma membrane	8	*ITGA2B*/*SELP*/*THBS1*/*CCR7*/*IL7R*/CXCR6/ABCB1/TRGV9	0.000330703	7.13771e − 06
GO:0034774	Secretory granule lumen	7	DEFA1B/HP/LTF/RNASE2/*THBS1*/PPIA/PRDX4	0.000330703	1.26687e − 05
GO:0060205	Cytoplasmic vesicle lumen	7	DEFA1B/HP/LTF/RNASE2/*THBS1*/PPIA/PRDX4	0.000330703	1.37218e − 05
GO:0031983	Vesicle lumen	7	DEFA1B/HP/LTF/RNASE2/*THBS1*/PPIA/PRDX4	0.000330703	1.4275e − 05
GO:0030877	*β*‐Catenin destruction complex	2	*CTNNB1*/AXIN2	0.007608835	0.000383179
GO:0097440	Apical dendrite	2	OSBP2/*SLC4A1*0	0.013830144	0.00087997
GO:0030667	Secretory granule membrane	5	*ITGB3*/*ITGA2B*/*SNCA*/*SELP*/GAA	0.013830144	0.000895477
GO:0071682	Endocytic vesicle lumen	2	HP/LTF	0.015142071	0.001089358

GO‐MFs					
GO:0019956	Chemokine binding	3	*ITGB3*/*CCR7*/CXCR6	0.014954812	9.22443e − 05
GO:0004896	Cytokine receptor activity	4	*CCR7*/*IL7R*/CXCR6/IL18RAP	0.014954812	0.000129479
GO:0019955	Cytokine binding	4	*ITGB3*/*THBS1*/*CCR7*/CXCR6	0.017978753	0.000459091
GO:0140375	Immune receptor activity	4	*CCR7*/*IL7R*/CXCR6/IL18RAP	0.017978753	0.000459091
GO:0050840	Extracellular matrix binding	3	*ITGB3*/*ITGA2B*/*THBS1*	0.017978753	0.000472924
GO:0005452	Inorganic anion exchanger activity	2	*SLC4A1*/*SLC4A1*0	0.017978753	0.000534603
GO:0046624	Sphingolipid transporter activity	2	MFSD2B/ABCB1	0.017978753	0.000622641
GO:0070411	I‐SMAD binding	2	*CTNNB1*/AXIN2	0.017978753	0.000622641
GO:0030246	Carbohydrate binding	5	PLOD2/*SELP*/LGALSL/GAA/KLRG1	0.018217271	0.000709764
GO:0015106	Bicarbonate transmembrane transporter activity	2	*SLC4A1*/*SLC4A1*0	0.030703573	0.001419821

**Table 3:** Top 10 KEGG pathway enrichment results for female‐specific DEGs. The analysis was performed on 58 female‐specific DEGs derived from 26 total samples (17 PAH females and nine female controls) using the limma package with an adjusted *p* value (FDR) < 0.05 as the significance threshold.

ID	Description	Count	Gene ID	*p* adjust	*p* value
hsa05418	Fluid shear stress and atherosclerosis	4	*CTNNB1*/*ITGB3*/*ITGA2B*/NOS3	0.052033934	0.000875452
hsa05412	Arrhythmogenic right ventricular cardiomyopathy	3	*CTNNB1*/*ITGB3*/*ITGA2B*	0.052033934	0.001757948
hsa04512	ECM–receptor interaction	3	*ITGB3*/*ITGA2B*/*THBS1*	0.052033934	0.002577168
hsa05165	Human papillomavirus infection	5	*CTNNB1*/*ITGB3*/*ITGA2B*/*THBS1*/AXIN2	0.052033934	0.003355137
hsa04510	Focal adhesion	4	*CTNNB1*/*ITGB3*/*ITGA2B*/*THBS1*	0.052033934	0.00351973
hsa04640	Hematopoietic cell lineage	3	*ITGB3*/*ITGA2B*/*IL7R*	0.052033934	0.003599839
hsa04015	Rap1 signaling pathway	4	*CTNNB1*/*ITGB3*/*ITGA2B*/*THBS1*	0.052033934	0.003974769
hsa04151	PI3K‐Akt signaling pathway	5	*ITGB3*/*ITGA2B*/*THBS1*/NOS3/*IL7R*	0.052033934	0.004476037
hsa04611	Platelet activation	3	*ITGB3*/*ITGA2B*/NOS3	0.069805539	0.006755375
hsa05144	Malaria	2	*SELP*/*THBS1*	0.094441712	0.010729492

In GO analysis, the most enriched BPs included cell–matrix adhesion, innate immune response in mucosa, and hydrogen peroxide (H_2_O_2_) catabolic process (Figure 2a). In the CC category, the platelet alpha granule membrane, platelet alpha granule, and exterior side of the plasma membrane were significantly linked to PAH in females (Figure 2b). Notably, enriched MFs encompassed organic chemokine binding, cytokine receptor activity, and immune receptor activity (Figure 2c). Furthermore, KEGG analysis suggested nominal enrichment for pathways, such as fluid shear stress and atherosclerosis, arrhythmogenic right ventricular cardiomyopathy (ARVC), and extracellular matrix cell (ECM)–receptor interaction (Figure 2d). These signal pathways should be interpreted cautiously as hypothesis‐generating rather than as evidence of direct disease comorbidity.

Figure 2GO and KEGG analyses of DEGs between PAH and controls in females. (a–c) The results of the Top 10 annotations for DEGs in females. (d) The Top 10 results of KEGG pathway analysis of DEGs in females based on enrichment score.

**Figure:**
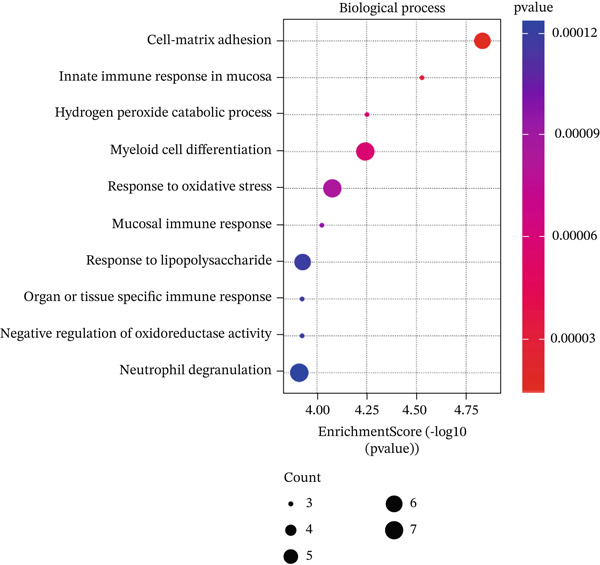
(a)

**Figure:**
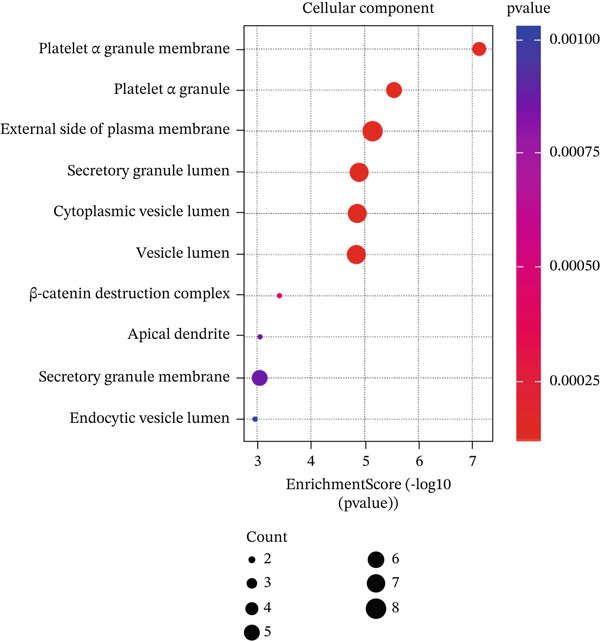
(b)

**Figure:**
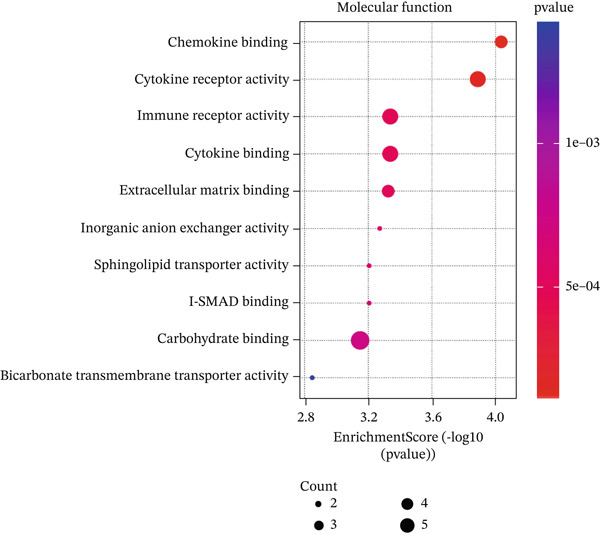
(c)

**Figure:**
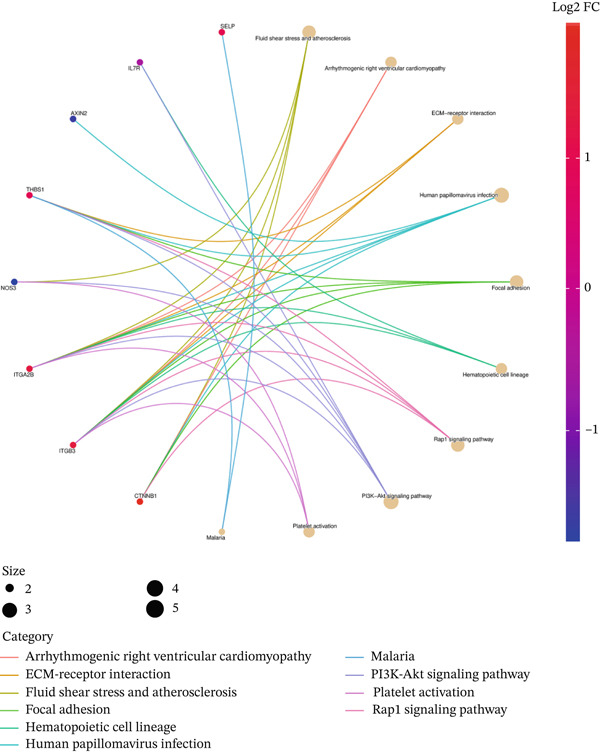
(d)

### 3.3. PPI Network Analysis and Hub Gene Identification

PPI network analysis was performed to study the relationships among DEGs in females. Fifty‐eight DEGs in females were uploaded to the STRING database, and only interactions with a score > 0.48 were selected (or the minimum connected network was selected). The Cytoscape software was used to visualize the resulting PPI network, which consisted of 34 nodes and 53 edges (Figure 3a). Subsequently, the CytoHubba plug‐in was employed to identify the most significant module using maximal clique centrality topology analysis methods. The Top 10 nodes with the highest connectivity in the network were identified as hub genes for further analysis: *CTNNB1*, solute carrier family 4 member 1 (*SLC4A1*), *SNCA*, *SELP*, *THBS1*, integrin beta‐3 (*ITGB3*), integrin alpha‐2b (*ITGA2B*), *CCR7*, *GZMK*, and interleukin‐7 receptor (*IL7R*). Moreover, the hub genes associated with cell–matrix adhesion, platelet degranulation, and blood coagulation were indicated by the most substantially enriched BPs. The changes of CCs containing platelet alpha granule membrane, platelet alpha granule, and the external side of the plasma membrane. MFs were mainly enriched in extracellular matrix binding, cytokine binding, and fibroblast growth factor binding (Figure 3b). Details of the hub genes, including their pathways, functions, and related disorders, are presented in Table 4.

Figure 3(a) The PPI network of DEGs in females, established using STRING and Cytoscape, contains 34 nodes and 53 edges. In this network representation, each node represents a gene, and the edges signify the molecular interactions between them. Upregulated genes are shown in red, and downregulated genes are shown in blue. Hub genes are highlighted within circles. (b) The Top 10 GO terms of the selected hub genes.

**Figure:**
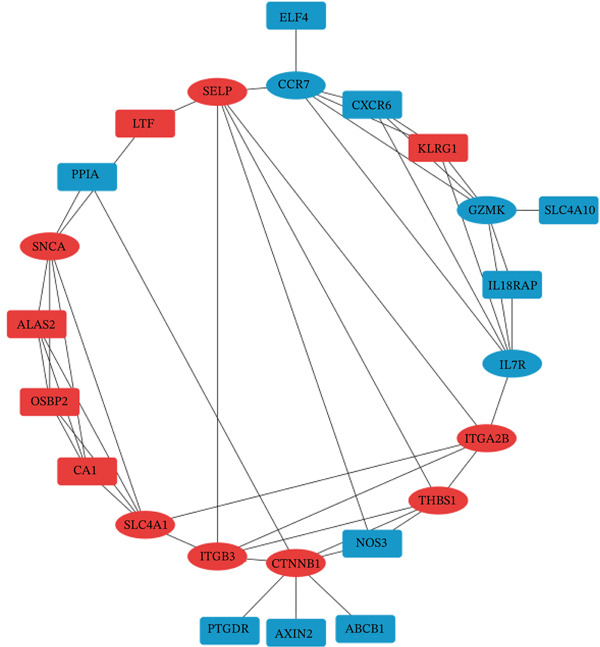
(a)

**Figure:**
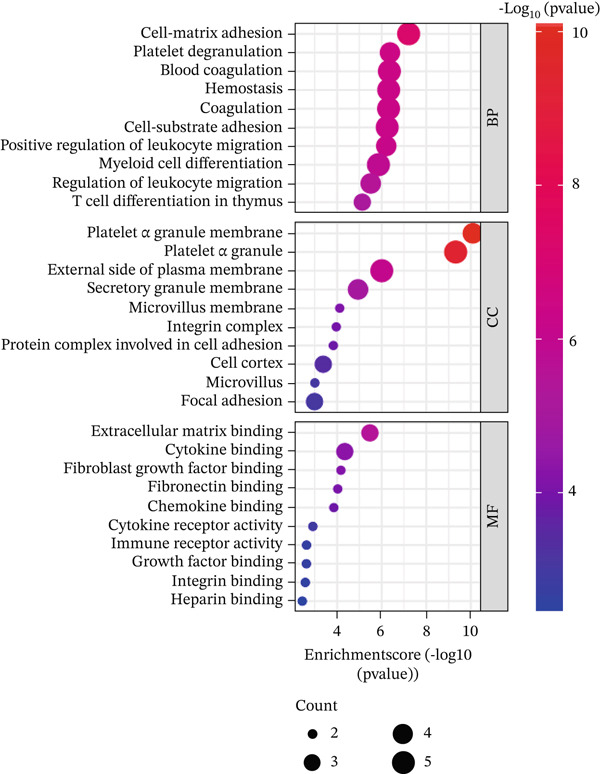
(b)

**Table 4:** Characteristics of hub genes: Pathways, functions, related disorders, and drug associations. This analysis was performed on 10 hub genes identified from 58 female‐specific DEGs derived from 26 total samples (17 PAH females and nine female controls). All enrichment analyses were conducted using an adjusted *p* value (FDR) < 0.05 as the significance threshold.

Gene′s name		Pathway	Function (GeneCards)	Disease (OMIM)	Drug (database source)
*CTNNB1* 	Estrogen‐induced Wnt/*β*‐catenin signaling and unbalanced estrogen signaling contribute to endometrial hyperplasia and cancer [21].	1. Wnt/*β*‐catenin signaling as a key pathway contributes to female differentiation [22].	1. Key downstream component of the canonical Wnt signaling pathway.Involved in the regulation of cell adhesion.A negative regulator of centrosome cohesion.	1. Ovarian cancer, somatic2. Medulloblastoma, somatic3. Neurodevelopmental disorder	1. Dexamethasone2. Thalidomide3. Cyclophosphamide (ClinPGx)
*SLC4A1* 	lnc‐*SLC4A1*‐1 recruited NF‐*κ*B to upregulate CXCL8 and induced the release of TNF‐*α* and IL1*β* following the activation of the inflammation response in trophoblast cells, which probably has a role in the URPL (a common pathological problem in pregnancy) [23].	1. *SLC4A1* is expressed on the erythrocytes and plays a significant role in the respiratory system.2. OS affects the binding of *SLC4A1* and spectrin, actin, by the ankyrin bridge.3. Interaction of *SLC4A1* and hemoglobin. Reactive oxygen species (ROS) can induce the generation of methemoglobin and glycated hemoglobin.4. lnc‐*SLC4A1*‐1 with NF‐*κ*B mediate the upregulation of CXCL8, which initiates inflammation. Activation of the NF‐*κ*B signaling pathway leads to the development of ALI [24].	1. Transporter mediates the 1:1 exchange of inorganic anions through the erythrocyte membrane [24].2. Biomarker of PAH, which can indicate an increased activation of some immune cells in their peripheral blood [24].	1. Hemolytic anemia, DRTA42. Ovalocytosis Southeast Asian3. Spherocytosis Type 4: SPH44. Acute lung injury (ALI) leading to pulmonary epithelial dysfunction and macrophage activation5. Pulmonary arterial hypertension (PAH) [24]	1. Atenolol2. Metoprolol(ClinPGx)
*SNCA* 	It is assumed that estrogen‐like activity of the *A. blazei* extract could induce an antioxidative effect due to the upregulation of GPX3 and *SNCA* expression in an ER‐dependent manner by estrogen [25].	1. The proteolytic role of Parkin in the ubiquitin‐proteasomal pathway.2. Induced intracellular signaling.3. Parkinson′s disease pathway.	1. Neuronal protein is involved in various synaptic activities, including the regulation of synaptic vesicle trafficking and the subsequent release of neurotransmitter.2. Acts by enhancing the release of local Ca (2+) from microdomains, a crucial process for the enhancement of ATP‐induced exocytosis.	1. Parkinson′s disease 42. Autosomal dominant; PARK4	1. Atropine2. Ketoconazole(ClinPGx)
*CCR7* 	*CCR7* is negatively correlated with estrogen receptor and progesterone receptor [22, 26].	1. The chemokine receptor *CCR7* regulates lymphocyte trafficking. Deficiency of *CCR7* leads to the infiltration of T and B cells in close proximity to vessels in the mouse lungs [27].	1. *CCR7* plays a role in immune surveillance and regulation of leukocyte movement [27].2. Acts as a receptor for the MIP‐3‐beta chemokine. Probable mediator of EBV effects on the lymphocyte function [27].	1. Role in pathogenesis of PAH. Perivascular infiltration of T and B cells in human pulmonary arterial hypertension is related to the downregulation of the *CCR7* receptors [27]	1. Tacrolimus2. Imatinib(GeneCards)
*GZMK* 	NF	1. Activate the protease‐activated receptor‐1 (PAR‐1) in endothelial and fibroblast cells.2. Induces the production of inflammatory cytokines.3. Affects the degradation of the extracellular matrix [28].	1. *GZMK* activates PAR‐1 in endothelial and fibroblast cells, leading to the stimulation of inflammatory cytokines, such as TNF‐*α*, IL1, IL‐6, and MCP‐1 in human infectious diseases. Additionally, the protease activity of *GZMK* can enhance the degradation of the extracellular matrix, consequently promoting inflammatory cell infiltration and tissue damage [28].	1. Tiggers the continuous inflammation amplification of rheumatoid arteritis [28]	1. Oxygen2. Serine(GeneCards)
*SELP* 	NF	1. Cell surface interactions at the vascular wall.2. Hemostasis.3. Platelet activation, signaling, and aggregation.4. Platelet degranulation.5. Response to elevated platelet cytosolic Ca^2+^.6. Immune response_IL‐12 signaling pathway.	1. Promotes the proliferation of the pulmonary artery′s smooth muscle cells, apoptosis resistance through increased oxidative stress, and mitochondrial dysfunction through activated hypoxia‐induced factor‐1*α* and metabolic dysfunction of glutathione.2. Increases quantity of M2‐polarized macrophages results in a decrease in *SELP*.3. The absence of *SELP* in a cancer cell culture elevates the amount of stem cells, indicating a rise in proliferative activities such as the generation of reactive oxygen species (ROS) and DNA damage. Oxidative stress leads to reduced cell survival and alters the Wnt pathway′s signals [29].	1. *SELP* contributes to the development of pulmonary hypertension [29]	1. Reviparin (GeneCards)2. Acetylsalicylic acid (DSigDB)3. Amiloride (GeneCards)
*IL7R* 	Estrogen has a suppressive effect on *IL7R* expression. This is because letrozole treatment increases the expression of the *IL7R* [30].	1. Rule in regulation, development, differentiation, and survival of T cells. 2. Apoptosis‐related network due to changed Notch3 in ovarian cancer [31].	1. *IL7R* is crucial to the development of T cells and immune competence.2. Receptor for interleukin‐7 and thymic stromal lymphopoietin.3. Known as a vital growth factor for the early lymphocyte development and supporting the growth of malignant cells in some lymphomas and leukemias.	1. Immunodeficiency (Type 2 diabetes)2. Chronic obstructive pulmonary disease3. Rheumatoid arthritis4. Associated with the survival of ovarian cancer patients	1. Heparin, bovine2. Herbimycin A(GeneCards)
*ITGA2B* 	NF	1. *ITGA2B* has an important role in normal platelet function by producing a key receptor in platelet aggregation (GPIIb) [32].	1. Galactosyltransferase activator, calmodulin‐regulated cell cycle (division) control‐related protein kinase.2. Integrin alpha‐2B, a cell surface adhesion receptor mediating cell adhesion to extracellular matrix or to other cells.	1. Bleeding disorder2. Glanzmann thrombasthenia 1; GT13. Arrhythmogenic right ventricular cardiomyopathy (ARVC)	1. Tirofiban (ClinPGx)2. Acetylsalicylic acid (GeneCards)3. Abciximab (GeneCards)
THBS1 	*THBS1* has protective effects of estrogen on vascular endothelial cells and vital roles in hydrogen peroxide–induced HUVEC cell injury and verification of estrogen′s impact on the expression levels of *THBS1* [33].	1. Inhibiting the ID1 target genes, such as *THBS1*, will raise the TSP‐1 protein levels, induce apoptosis in pulmonary endothelial cells, and stimulate proliferation in smooth muscle cells [34].2. TSP‐1 has been linked to the activation of TGF‐*β*, modulation of nitric oxide (NO) signaling, and augmentation of reactive oxygen species (ROS) production, all pathways implicated in the molecular pathogenesis of pulmonary arterial hypertension (PAH) [34].3. *THBS1* is involved in the cellular adhesion signaling pathway as an adhesive glycoprotein that regulates cell‐ECM/cell interactions. Downregulation of *THBS1* in ovarian cancer promotes the migration of the tumor [35].4. TGF‐*β* pathway.	1. Contributes to inflammation, wound healing, angiogenesis, reactive oxygen species (ROS) signaling, nitrous signaling, aging, apoptosis, senescence, stemness, cellular self‐renewal, and cardiovascular and metabolic homeostasis.2. Induced in arteries and lung parenchyma following injury or stress. The 362Asn *THBS1* mutation, as a missense alteration, has been preserved in the TSR1 region. TSRs have roles in activating latent TGF‐*β*1, restraining smooth muscle cell proliferation, and serving as inhibitors of endothelial cell growth, making this mutation important [36].	1. *THBS1* and *THBS2* exhibit a correlation with pulmonary arterial hypertension (PAH) and an unfavorable prognosis in individuals diagnosed with heart failure	1. Cyclophosphamide2. Cyclosporine(GeneCards)
*ITGB3* 	As a result of treating a subset of OSCs (*n* = 3) with estrogen and hypoxia for 48 h, the level of *ITGB3* mRNA increased [37].	1. Known as the platelet glycoprotein IIIa and antigen integrin *β*3 (CD61), it is modulated by miR‐95 to regulate the migration, proliferation, and invasion of non–small‐cell lung cancer, which is a pathological feature of PAH [38].	1. *ITGB3* improves the proliferation of PASMCs and verifies the suppression of *ITGB3* to decrease the progression of PAH [38].2. Integrin alpha‐V/beta‐3 (ITGAV: *ITGB3*) is a receptor for cytotoxin, fibronectin, thrombospondin, vitronectin, prothrombin, and von Willebrand factor.3. Integrin ITGAV: *ITGB3* acts as a receptor for the herpesvirus.	1. Bleeding disorder2. Platelet‐Type, 24; BDPLT243. Glanzmann thrombasthenia 2; GT24. *ITGB3* remarkably promotes the development of PAH [38]	1. Acetylsalicylic acid2. Clopidog (ClinPGx)

### 3.4. Identification of Chemicals Related to Hub Genes

**Figure 4:**
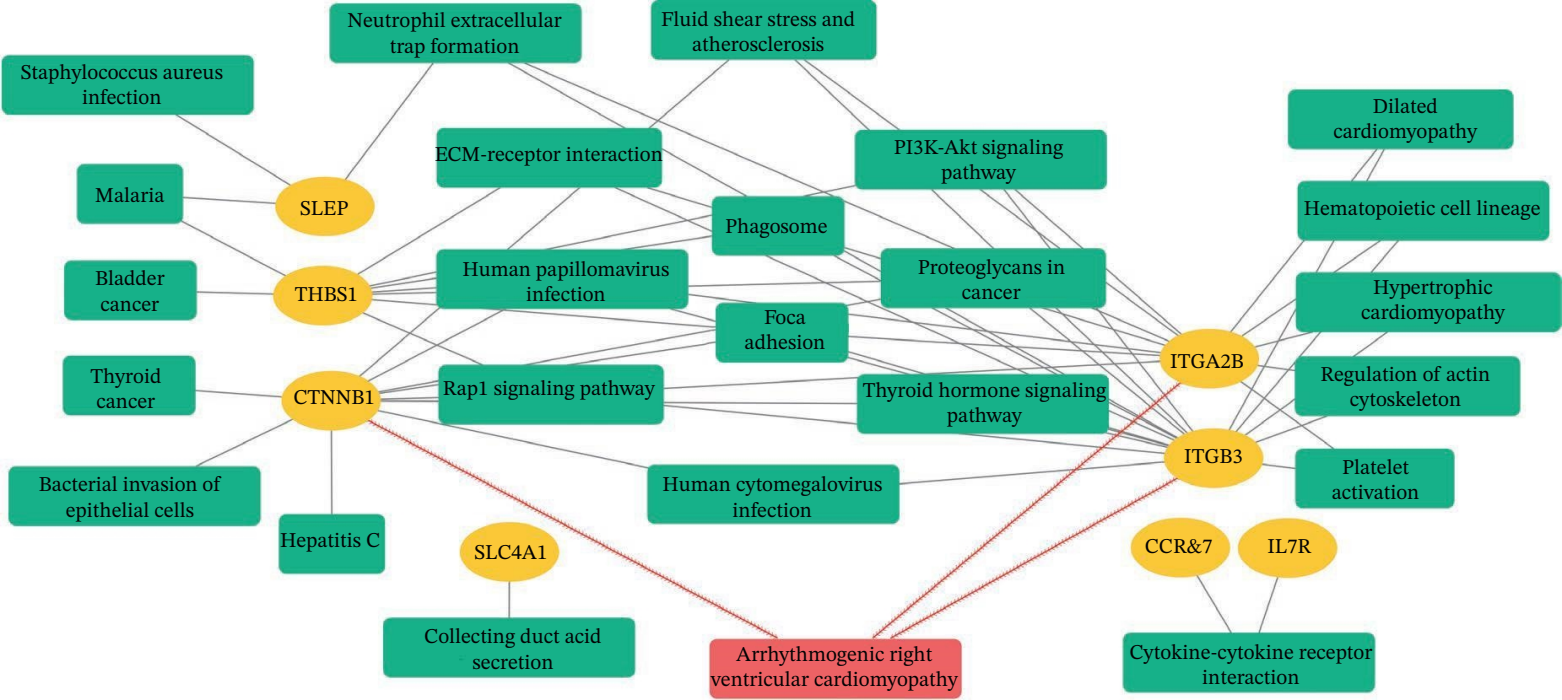
A detailed network of hub genes enriched in related pathways. Orange ovals represent hub genes within the network. The most related pathway was arrhythmogenic right ventricular cardiomyopathy, as highlighted by the connections to the hub genes.

In order to predict the compounds that may affect the expression of each central gene in PAH, we evaluated each gene. To identify potential therapeutic compounds that may modulate the expression of hub genes in PAH, a multistep pharmacogenomic approach was applied. Initially, enrichment analysis was performed using the Enrichr platform (https://maayanlab.cloud/enrichr/) and its Drug Signatures Database (DSigDB) module, which integrates experimentally validated drug–target interactions and gene expression perturbations. Compounds were prioritized based on enrichment scores and adjusted *p* values (FDR < 0.05). Among the top negatively enriched results, acetylsalicylic acid (aspirin) was identified as inversely correlated with *SELP*, suggesting an anti‐inflammatory and antiplatelet role in pulmonary vascular remodeling. To further validate these associations, clinically curated and literature‐based pharmacogenomic resources were consulted. Specifically, ClinPGx (https://clinpgx.com) confirmed the following gene–drug associations: *CTNNB1* (dexamethasone, thalidomide, and cyclophosphamide), *SLC4A1* (atenolol and metoprolol), *ITGB3* (acetylsalicylic acid and clopidogrel), and *ITGA2B* (tirofiban). In addition, complementary validation for the remaining hub genes and their associated compounds was obtained from the GeneCards database (https://www.genecards.org/), which integrates curated data from DrugBank, DGIdb, PharmGKB, and Novoseek. This integrative approach—combining DSigDB for transcriptomic prediction, ClinPGx for clinical validation, and GeneCards for multisource curation—provides a comprehensive framework for identifying potential pharmacological modulators of PAH‐related hub genes. Details of these genes and validated drug interactions are summarized in Table 4, while the enriched network indicates that ARVC remains the most significantly associated pathway (Figure 4).

## 4. Discussion

PAH is a progressive disorder shaped by environmental, genetic, and epigenetic influences, yet its underlying molecular pathways remain only partly understood [39]. It is generally established that PAH is more prevalent in women than in men. Despite a higher frequency, men often have worse survival rates. Estrogen and other female sex hormones have been suggested to have a role in the progression of PAH despite treatment; nevertheless, the impact of sex hormones on the fundamental pathophysiology remains contentious [40]. Currently, there is no specific strategy to treat PAH, and treatment focuses on managing symptoms and improving quality of life. Identifying biomarkers and underlying mechanisms associated with gender‐specific factors can improve our abilities to develop strategies for screening, early diagnosis, and treatment of PAH. The rapid advancement of high‐throughput expression technology alongside bioinformatics has the potential to offer additional resources for identifying diagnostic biomarkers and providing prognostic insights for various diseases.

Two microarray datasets, GSE38267 and GSE131793, were studied, and the DEGs were separately determined by bioinformatic approaches. The results showed that GSE38267 and GSE131793 were involved in 718 and 170 DEGs in PAH females, respectively (Figure 1). Based on logFC and *p* values, 34 downregulated and 24 upregulated genes were selected as DEGs in females. To obtain a deeper understanding of the function of the selected DEGs in PAH females, GO and KEGG analyses were carried out (Tables 2 and 3). Furthermore, the STRING database and Cytoscape software were employed to conduct a PPI network analysis of 58 female‐specific DEGs, resulting in the identification of 10 hub genes. Our results indicated that *CTNNB1*, *SLC4A1*, *SNCA*, *SELP*, *THBS1*, *ITGA2B*, and *ITGB3* genes were upregulated in PAH females, while *CCR7*, *GZMK*, and *IL17R* genes were downregulated in PAH females (Figure 3a). However, the role of some of the identified hub genes in PAH pathogenesis was published in the literature previously.

The *CTNNB1* gene is responsible for encoding *β*‐catenin protein, which plays a crucial role in the Wnt signaling pathway. Our findings align with prior research indicating that the Wnt signaling pathway may significantly influence the development and progression of PAH, especially with sex‐related variations. This outcome may explain the elevated incidence of PAH in women and the enhanced long‐term survival rates among female patients [12, 41]. Somatic variants of the *CTNNB1* gene were identified in colorectal and ovarian cancer. Furthermore, studies showed that deleting the *CTNNB1* gene in mice during embryonic development can lead to the inhibition of lung progenitor specification and tracheal budding, ultimately resulting in respiratory failure in terms of impaired formation and differentiation of the peripheral lung [16].

On the other hand, it was indicated that *β*‐catenin may play a significant role in pulmonary artery endothelial‐mesenchymal transformation in rats with chronic thromboembolic pulmonary hypertension (CTEPH). Furthermore, inhibiting *β*‐catenin could potentially offer therapeutic strategies for CTEPH [42]. Our results indicate that the Wnt signaling pathway may be responsible for the sex‐related variations in PAH. Nevertheless, this observation should be regarded as exploratory. The potential role of the *CTNNB1* gene in PAH pathogenesis may be suggested by the increased expression of the gene in females. However, additional experimental studies will be necessary before it can be considered a predictive indicator or therapeutic target. Thus, our results indicated an association between this gene and ARVC, consistent with previous studies (Figure 4).

In recent years, there has been increasing evidence suggesting that the canonical Wnt/*β*‐catenin pathway played a role in the molecular development of arrhythmogenic cardiomyopathy [43]. Studies showed that the Wnt/*β*‐catenin/Tcf/Lef pathway is suppressed in AC in terms of the nuclear translocation of plakoglobin. Plakoglobin, also known as *γ*‐catenin, shares a high similarity with *β*‐catenin, which serves as the effector of the canonical Wnt signaling pathway [44, 45]. *CTNNB1* may be modulated by estrogen signaling, which can influence endothelial cell proliferation and smooth muscle differentiation. Such hormone‐dependent modulation could partly explain the higher incidence but improved survival among female PAH patients. One of the obtained hub genes in this study is *SLC4A1*, which is a member of the anion exchanger family. It plays a significant function in respiratory systems, like the Jacobs–Stewart cycle, and is mostly expressed on erythrocytes. Because it has the ability to move carbon dioxide from tissues to the lungs and change chloride into bicarbonate in the erythrocyte membrane, it is crucial for efficient respiration [46]. In a genome‐wide association study, it was determined that *SLC4A1* has a significant role in the regulatory structure of erythrocytes [40]. Moreover, in several studies that did not specifically focus on sex differences, *SLC4A1* was recognized as the hub gene in PAH pathogenesis [41, 47]. The higher *SLC4A1* expression observed in females may also reflect sex‐dependent erythrocyte metabolism and oxygen transport differences, potentially influenced by hormonal regulation. We suggest that this stems from the high overexpression of *SLC4A1* observed in females across all of these studies.

The other hub gene is the *SNCA* gene from the synuclein family, including *α*‐ and *β*‐synuclein, and encodes the *α*‐synuclein protein. However, synuclein is expressed in the cerebrum, which selectively inhibits phospholipase D2. It is reported that *SNCA* expression was significantly reduced in lung adenocarcinoma (ADC) [48], while our results showed that this gene was upregulated in the female PAH compared to the healthy control. Additionally, the *SNCA* gene was identified in KEGG pathways that were associated with Parkinson′s disease. There is no direct mechanistic link implied by this enrichment, which should be interpreted with caution. Hypotheses are generated by such associations, necessitating additional research to determine whether there is any biological significance between *SNCA* and PAH [38]. Given evidence that estrogen modulates *α*‐synuclein expression in neuronal and endothelial tissues, the upregulation of *SNCA* in females may similarly reflect sex hormone–driven transcriptional control.

Also, our analysis indicated that the expression of the *SELP* gene was upregulated in female PAH patients. The *SELP* gene encodes a protein called P‐selectin, which is an adhesion molecule expressed on the surface of activated platelets and endothelial cells [49]. The elevated P‐selectin expression can lead to the enhanced recruitment and adhesion of inflammatory cells to the pulmonary artery walls. The recruited inflammatory cells, such as leukocytes, have the ability to produce several proinflammatory mediators. This process further enhanced inflammation, vasoconstriction, and the restructuring of pulmonary arteries [50]. In addition to its role in inflammation, P‐selectin was implicated in the regulation of endothelial cell function and the release of certain molecules involved in vascular homeostasis. Endothelial dysfunction is a hallmark of PAH and contributes to the pathological changes in the pulmonary arteries. The dysregulation of P‐selectin expression and function in PAH may disrupt normal endothelial cell function, further exacerbating the disease [51, 52]. Increased *SELP* expression in females may also be related to estrogen‐mediated enhancement of endothelial activation and leukocyte adhesion, consistent with sex‐specific vascular inflammatory responses. These results suggest that anti‐P‐selectin tactics may be investigated in further research. However, without experimental confirmation, our bioinformatic data is not enough to support therapeutic repurposing.

The *THBS1* gene encodes a protein called thrombospondin‐1, which is an important regulator of various cellular processes, including cell adhesion, migration, proliferation, and angiogenesis [46]. Buda et al. have reported that the expression of thrombospondin‐1 is significantly upregulated in the pulmonary vasculature of PAH patients. In patients with PAH, elevated levels of thrombospondin‐1 were seen in the vascular smooth muscle and endothelial cells of the pulmonary arteries [53]. The increased thrombospondin‐1 in PAH is believed to contribute to the disease pathogenesis via several mechanisms. Firstly, thrombospondin‐1 can promote endothelial dysfunction, leading to impaired vasodilation and increased vasoconstriction of the pulmonary arteries. Secondly, it can stimulate the proliferation and migration of vascular smooth muscle cells, contributing to pathological vascular remodeling and narrowing of the pulmonary arteries. This vascular remodeling further elevates pulmonary vascular resistance, a hallmark of PAH [54]. According to Zeng et al., there is a considerable upregulation of the *THBS1* gene in hypertension circumstances [55]. Their findings demonstrated that elevated thrombospondin‐1 levels were linked to the inhibition of angiogenesis and vascular growth, which exacerbated the rise in blood pressure. Thrombospondin‐1 was found to play a role in various biological pathways that are associated with the development of PAH. These pathways include TGF‐*β* activation, regulation of nitric oxide (NO) signaling, and promotion of reactive oxygen species (ROS) [34]. In our study, it was observed that the *THBS1* gene is upregulated in PAH females, which is compatible with previous investigations and may indicate an increased production of thrombospondin‐1 protein in female PAH patients.

The other hub gene, *ITGA2B*, is a protein that forms a subunit of the integrin *α*IIb*β*3 receptor, primarily expressed on platelets. Although *ITGA2B* is not directly linked to PAH development, it has been implicated in several features of the illness. Furthermore, *ITGA2B* mutations may cause platelet‐type bleeding illnesses such as Glanzmann thrombasthenia, which is characterized by platelet failure to aggregate [56]. Some studies suggested that platelet activation and abnormal aggregation may occur in PAH, which led to the formation of small blood clots within the pulmonary vasculature. Therefore, these clots can obstruct blood flow and contribute to the development and progression of PAH [41, 57]. We observed the upregulation of the *ITGA2B* gene expression in the females with PAH. The upregulation of *ITGA2B* expression could potentially contribute to increased platelet activation and aggregation, leading to the prothrombotic state observed in PAH.

*ITGB3* is a protein that forms a subunit of the integrin *α*v*β*3 receptor, which is involved in cell adhesion, angiogenesis, cell migration, extracellular matrix remodeling, and signaling processes [58].

It is well approved that the expression of *ITGB3* increases in the smooth muscle cells of pulmonary arteries in PAH patients. The upregulation of *ITGB3* suggests that it may be involved in the abnormal development and migration of vascular smooth muscle cells. These cells play a crucial role in the development of pulmonary vascular remodeling, a hallmark of PAH [59]. Furthermore, we observed increased *ITGB3* expression in female PAH patients, suggesting that this upregulation could alter cell–cell and cell–matrix interactions and modify signaling pathways involved in vascular remodeling, angiogenesis, and disease progression. This is consistent with a study conducted by Liu et al., who reported that *ITGB3* was upregulated in PAH samples compared to healthy controls using microarray analysis [38]. According to the study′s results, females with PAH had higher levels of *ITGB3* expression. In addition to altering signaling pathways that regulate vascular remodeling, angiogenesis, and other processes pertinent to the onset and progression of PAH, this upregulation of *ITGB3* may also result in changes in cell–cell and cell–matrix interactions. Besides, Pan et al. showed that *ITGA2B* and *ITGB3* were upregulated in stress cardiomyopathy [60]. Our results indicated that these genes map within pathways annotated as ARVC (Figure 4). This enrichment should be regarded as exploratory rather than evidence of a direct disease association (Figure 4). Given that platelet activation and extracellular matrix remodeling are influenced by hormonal balance, the upregulation of *THBS1* and *ITGB3* in females may represent an adaptive vascular response under estrogenic control, rather than a purely pathological event.

The *CCR7* gene is responsible for producing the CCR7 protein, which was detected as a hub gene in the PAH females. It is a member of the G protein‐coupled receptor family and is a chemokine receptor. This protein is essential for controlling lymphocyte trafficking. In mice, *CCR7* loss causes an increase in T and B lymphocytes near blood arteries in the lung. Our study showed that this gene was downregulated in PAH females compared to men and healthy controls. This result was comparable with those previously obtained by Larsen et al., who concluded that lower levels of *CCR7* can have a role in the development of PAH through secretion of CX3CL1 and IL‐12 and infiltration of lymphocytes [50, 61]. Moreover, a study showed an increase in *CCR7* expression in breast cancer [62]. However, there may be an association between *CCR7* expression and female sex hormones that needs further investigation. Similarly, *IL7R* downregulation in females could reflect distinct immune‐regulatory adjustments known to occur under estrogen‐dominant conditions, consistent with sex‐biased immune activation reported in PAH.

The *GZMK* gene encodes the granzyme K (GrK) protein, which belongs to the family of serine proteases known as granzymes and is a hub gene in this study. GrK is expressed by the granules of NK (natural killer) cells and CTLs (cytotoxic T lymphocytes). Prior research has shown that *GZMK* has the ability to trigger nonapoptotic cell death by generating ROS and disrupting mitochondrial function when paired with perforin [63]. Therefore, *GZMK* is mostly associated with fibroblasts and endothelial cells, suggesting that it plays a role in rheumatoid arthritis′s aberrant angiogenesis and synovial hyperplasia [53]. Our findings showed that, in contrast to healthy females and males, the *GZMK* gene expression is downregulated in female PAH patients. This result was in contrast with those previously obtained by Zeng et al., who indicated that the expression of *GZMK* was upregulated in the PAH samples [55]. Considering the usage of female and male samples in their study, we proposed that the expression of *GZMK* may exhibit significant differences between female and male individuals with PAH. The observed downregulation of *GZMK* in females could reflect sex‐linked differences in cytotoxic lymphocyte activation, consistent with prior reports of attenuated NK cell cytotoxicity under estrogen dominance.

*IL7R*, known as a hub gene, is a protein‐coding gene that plays a significant role in the immune system. It is primarily expressed on the surface of immune cells, including T cells and B cells [31]. Dysregulation of IL‐7 signaling was linked to various autoimmune diseases and inflammatory conditions [56]. While the specific mechanisms behind PAH development are not fully understood, it is hypothesized that immunological dysregulation and inflammatory processes have a role in the remodeling of the pulmonary vasculature, resulting in higher pulmonary artery pressure [21]. Our results showed a downregulation in the expression of the *IL7R* gene in females with PAH.

DEGs associated with the GO BP class primarily include cell matrix adhesion, innate immune response in mucosa, and the H_2_O_2_ catabolic process (Figure 2a). Cell–matrix adhesion plays a critical role in maintaining the structural integrity of tissues and organs in the body by facilitating the interaction between cells and the extracellular matrix [64]. It is widely recognized that one of the major causes of PAH is the remodeling of the pulmonary artery′s extracellular matrix, which promotes collagen deposition. In fact, an imbalance between proteolytic enzymes and their natural tissue inhibitors led to extracellular matrix remodeling. Thus, the initiation of pulmonary vascular remodeling in PAH is largely dependent on this remodeling. Increased hypoxia, inflammation, TGF‐*β*, and altered BMPR2 signaling are some of the elements that define the pathophysiology of pulmonary vascular extracellular matrix remodeling in PAH [59, 65]. Considering the importance of extracellular matrix remodeling in the pathogenesis of PAH, it has the potential to be a therapeutic target. On the other hand, pulmonary vascular remodeling in patients with PAH is often associated with perivascular inflammation. These immune cells, such as macrophages, dendritic cells, and T and B lymphocytes, were shown to have a substantial impact on the process of pulmonary vascular remodeling in most cases of PAH. Therefore, the GO BP data obtained demonstrated that the catabolic process of H_2_O_2_ plays a significant role in PAH illness. In inflammatory diseases such as PAH, mitochondrial ROS are significant. In both physiological and pathological processes, ROS are essential. ROS includes radicals with unpaired electrons, such as H_2_O_2_. In addition to its role as an intracellular mediator, H_2_O_2_ has a variety of effects on endothelial cell function, including the regulation of inflammatory responses. The maintenance of vascular homeostasis necessitates the controlled regulation of H_2_O_2_. However, abnormal redox signaling, often caused by excessive ROS production and/or decreased antioxidant activity, can disrupt vascular function and contribute to vascular diseases [66, 67]. Moreover, the results of the CC class demonstrated that DEGs were enriched in the external side of the plasma membrane, platelet alpha granule membrane, and platelet alpha granule (Figure 2b). Platelet alpha granules are a valuable source of growth factors, cytokines, and chemokines. Consequently, they are essential for immune responses and inflammatory reactions [68, 69]. Notably, enriched MFs consisted of organic chemokine binding, cytokine receptor activity, and immune receptor activity (Figure 2c). It is widely recognized that the PAH is not exclusively the result of dynamic vasoconstriction. In addition, inflammation and immune cells are critically involved in the pathophysiology and progression of PAH. Chemokines in particular were shown to contribute significantly to pulmonary artery remodeling. In a healthy pulmonary artery, the level of inflammatory chemokines is typically low, which aids in the preservation of local vascular homeostasis. Nevertheless, the expression of a variety of chemokines in the endothelial cells, smooth muscle cells, and fibroblasts of the pulmonary artery can be induced by a variety of factors, including hypoxia, shear stress, toxins, and genetic predisposition [61]. Nevertheless, in PAH, immune cells migrate into the pulmonary vasculature walls and induce an increase in cytokine and chemokine levels in the circulation and the adjacent tissues of the pulmonary vessels [29]. Our analysis of Go MF in female PAH patients points exactly in this direction. Consequently, new approaches such as immunotherapy and anti‐inflammatory treatments may be proposed to halt the progression of PAH.

The enriched KEGG pathways of DEGs in females indicate that focal adhesion, ARVC, the Rap1 signaling pathway, ECM–receptor interaction, and fluid shear stress and atherosclerosis might be related to the development of PAH (Figure 2d). Focal adhesions are significant protein complexes that develop between the plasma membrane and the ECM. They play an important role in cell–matrix interactions and have a significant impact on cell behavior, including differentiation, proliferation, and migration [70]. The focal adhesion kinase (FAK) family controls focal adhesion dynamics via integrins [63, 71]. It was proven that there is an association between FAK and vascular diseases [72]. Reports showed that in the PAH, FAK can stimulate the proliferation of pulmonary arterial smooth muscle cells [73]. As a result, FAK inhibition may be beneficial in improving functionalities associated with monocrotaline‐induced PAH in vivo, such as hemodynamics, vascular remodeling, and right ventricular hypertrophy. Thus, FAK may be considered a novel treatment target for PAH. Rap1 is a GTPase belonging to the Ras superfamily, which is linked to various cellular functions. Recent studies are highlighting the crucial role of Rap GTPase in the cardiovascular system. Within this system, Rap effector proteins are involved in pathways that regulate cell adhesion, proliferation, and migration, ultimately contributing to the regulation of cardiovascular function [74].

Rap1b has been shown to play an important role in blood pressure regulation by modulating vascular tone in smooth muscle and endothelial cells. This impact affects a variety of cell types, including hematological, endothelial, smooth muscle, and cardiac myocyte cells [75].

While several identified hub genes, including *CTNNB1*, *THBS1*, *ITGB3*, and *SLC4A1*, have been previously associated with PAH pathogenesis, the originality of our analysis resides in the demonstration that these genes are distinctly dysregulated in females, but not in men, across two separate datasets. This female‐specific signature highlights sex‐dependent molecular mechanisms that are often overlooked in PAH research. Moreover, hub genes, such as *SNCA*, *GZMK*, and *IL7R*, which have rarely been associated with PAH in prior studies, emerged here as female‐specific candidates. Thus, our work contributes novel insights by disentangling sex‐based differences at the transcriptomic level and by providing a prioritized set of genes for future experimental validation in female PAH patients. Taken together, these hub genes highlight sex‐dependent differences in vascular remodeling, immune regulation, and estrogen‐responsive pathways that may plausibly contribute to the higher susceptibility of females to PAH, although further experimental validation is needed.

## 5. Limitations

A limitation of our study is that, although within‐dataset normalization was applied, batch effects across datasets could not be fully eliminated because GSE38267 and GSE131793 were generated on different microarray platforms. We limited downstream analysis to genes that were consistently found in both female datasets in order to address this problem. However, it will be beneficial to conduct future research with bigger multicenter cohorts and batch‐effect correction techniques (e.g., ComBat or cross‐platform normalization) with standardized data processing. An additional strength of this study was that DEG identification and enrichment analyses were performed under stringent FDR control, which increased the robustness of findings. It is also important to note that some pathway enrichments (e.g., Parkinson′s disease and ARVC) are speculative and should be considered hypothesis‐generating rather than definitive; future experimental validation is essential. Another limitation of our study is the lack of experimental validation (e.g., qPCR or functional assays). As a result, even if our bioinformatic analysis found hub genes unique to women, their potential as biomarkers or therapeutic targets should be seen as speculative unless confirmed in other cohorts and lab tests.

## 6. Conclusions

Through a bioinformatic analysis of microarray datasets, we identified 10 female‐specific hub genes (*SLC4A1*, *THBS1*, *ITGB3*, *IL7R*, *CCR7*, *SNCA*, *CTNNB1*, *SELP*, *GZMK*, and *ITGA2B*) from DEGs between control and PAH samples after excluding male‐specific and shared DEGs. These genes may serve as intriguing targets for future exploration in PAH. The significance of our findings is in the demonstration that these hub genes are uniquely dysregulated in females, but not in men, hence underscoring a female‐specific molecular signature in PAH. However, given that our study was based solely on bioinformatic analyses without experimental validation, their potential utility should be regarded as preliminary and hypothesis‐generating, requiring confirmation in future laboratory and clinical studies. Our results highlight the need for directing future research toward sex‐dependent molecular processes to enhance the understanding of PAH pathophysiology and refine female‐specific diagnostic and treatment strategies.

## Author Contributions

Conceptualization: M.P., H.K., R.K., and M‐H.M. Methodology: M.P., M‐H.M., F.J.T., and and Z.S. Software: M‐H.M., F.J.T., Z.S., and M.P. Data collection: Z.S., R.K., F.J.T., M‐H.M., H.K., and M.P. Investigation: R.K., F.J.T., M‐H.M., H.K., M.P., and Z.S. Resources: F.J.T., R.K., M‐H.M., H.K., and M.P. Data curation: R.K., H.K., M‐H.M., and M.P. Writing—original draft preparation: M.P., M‐H.M., R.K., H.K., and F.J.T. Writing—review and editing: H.K., M.P., and R.K. Visualization: M‐H.M., F.J.T., and Z.S. Project administration: H.K. and M.P.

## Funding

No funding was received for this manuscript.

## Disclosure

All authors have read and agreed to the published version of the manuscript.

## Ethics Statement

This study exclusively analyzed publicly available, deidentified human microarray datasets from the NCBI GEO database (GSE38267 and GSE131793). No new human subjects were recruited, and no identifiable information was accessed. The initial investigations secured informed consent and local ethical permissions; our analysis adhered to GEO data use regulations.

## Conflicts of Interest

The authors declare no conflicts of interest.

## Data Availability

The data used to support the findings of this study are available from the corresponding author upon request.
